# A Comparative Analysis of Biosynthetic Gene Clusters in Lean and Obese Humans

**DOI:** 10.1155/2019/6361320

**Published:** 2019-06-12

**Authors:** Shengqin Wang, Na Li, Nan Li, Huixi Zou, Mingjiang Wu

**Affiliations:** ^1^College of Life and Environmental Science, Wenzhou University, Wenzhou 325035, China; ^2^Ruian Center for Disease Control and Prevention, Wenzhou 325035, China

## Abstract

Obesity is intrinsically linked with the gut microbiome, and studies have identified several obesity-associated microbes. The microbe-microbe interactions can alter the composition of the microbial community and influence host health by producing secondary metabolites (SMs). However, the contribution of these SMs in the prevention and treatment of obesity has been largely ignored. We identified several SM-encoding biosynthetic gene clusters (BGCs) from the metagenomic data of lean and obese individuals and found significant association between some BGCs, including those that produce hitherto unknown SM, and obesity. In addition, the mean abundance of BGCs was positively correlated with obesity, consistent with the lower taxonomic diversity in the gut microbiota of obese individuals. By comparing the BGCs of known SM between obese and nonobese samples, we found that menaquinone produced by* Enterobacter cloacae* showed the highest correlation with BMI, in agreement with a recent study on human adipose tissue composition. Furthermore, an obesity-related nonribosomal peptide synthetase (NRPS) was negatively associated with Bacteroidetes, indicating that the SMs produced by intestinal microbes in obese individuals can change the microbiome structure. This is the first systemic study of the association between gut microbiome BGCs and obesity and provides new insights into the causes of obesity.

## 1. Introduction

Recent studies show that gut microbes play an important role in the pathogenesis of obesity [1, 2]. Diet-induced alteration of the gut microbiota alleviated obesity in children [3, 4], and several intestinal microbes (e.g., Actinobacteria) have been significantly associated with obesity [5]. Occasionally, an obesity-associated microbe detected in one study cannot be validated in other studies. For example, some studies report an increased Firmicutes to Bacteroidetes ratio in obese patients [6, 7], whereas others found no association between the above phyla and obesity [8, 9].

Microbial interactions can alter the composition of the community by producing secondary metabolites (SMs). SMs are organic compounds that are produced by bacteria and fungi that can mediate microbial competition and interaction and therefore influence the composition of the gut microbiota. The biosynthesis of SMs is controlled by enzymes encoded by biosynthetic gene clusters (BGCs). Genomic mining of gut microbiota BGCs has helped identify numerous bioactive SMs with antimicrobial potential [10, 11]. Most microbial BGCs that have been identified so far contain genes encoding core biosynthetic enzymes such as polyketide synthase (PKS) and nonribosomal peptide synthetase (NRPS). More than 3000 small molecule BGCs were identified in the NIH Human Microbiome Project [12], of which lactocillin showed similar structure to some clinically tested antibiotics, and the* in vivo* expression was validated by the metatranscriptome sequencing analysis. These small molecules not only inhibit the growth of competing bacteria but also alter the composition of the gut microbiome. In addition, microbial SMs have also been implicated in human physiology, although their precise role in obesity is unclear.

In our previous study, we used a systematic approach to detect putative BGCs enriched in Parkinson's disease from raw metagenomic data, of which many originated from microbes that were not abundant in the corresponding patients [13]. In this study, we analyzed the differences in the BGCs of nonobese and obese individuals using human fecal metagenomic data, in order to identify obesity-associated BGCs. Our findings illustrate the widespread distribution of SM-encoding BGCs in the human microbiome and provide new insights into the causes of obesity.

## 2. Methods

### 2.1. Data Collection and Construction of Human-Related BGC Protein Database

A total of 10,042 genomes were identified from the IMG-ABC website (Version 4.560) using “Homo sapiens” as the host name, and 246,188 human-related BGCs and 2,640,191 protein sequences were extracted from these genomes [14]. The gut metagenomic data was extracted from the European Bioinformatics Institute-Sequence Read Archive database using the accession number ERP003612, which initially was used to analyze the correlation between the colonic microbiota and metabolic disorders in a Danish cohort of 123 nonobese and 169 obese individuals [15]. In the quality control step, we only kept the first 70bp of the reads for each sample, and samples with read length less than 70bp were discarded. The remaining 278 samples were analyzed further.

### 2.2. Identification of Putative BGCs

To determine the abundance of each putative BGC per sample, the metagenomic reads were first aligned against the protein sequence database of the human-related BGCs using the DIAMOND tool with an E-value of 1e-05 [16], and the top hit proteins per read were subsequently analyzed. To avoid contamination of the nonbiosynthesis genes, a list of biosynthesis and nonbiosynthesis related Pfam domains was, respectively, extracted from AntiSMASH [17] and a recent study [12]. A database was constructed using these Pfam domains and queried against the top hit proteins, and the biosynthesis genes were validated using the hmmscan program in the HMMER package with an E-value of 0.01. Finally, the abundance scores of the biosynthesis genes of each BGC per sample were calculated, and the BGCs with at least 50% biosynthesis genes that were detected in more than 10 samples with a frequency of reads ≥ 10 were selected for the following analysis [13].

### 2.3. Detection of Known SM-Encoding BGCs

A database of 13460 protein sequences extracted from all SM-encoding BGCs was constructed, and the trimmed metagenomic reads from ERP003612 were aligned against this database using the DIAMOND tool with an E-value of 1e-05 [16]. The putative BGCs encoding known SMs were detected the same way as the human-related BGCs. For each known secondary metabolite BGC, we compared their abundance with the body mass index (BMI).

### 2.4. Normalization and Comparison

The abundance of these putative BGCs and known SM-encoding BGCs was further assessed across different samples. Each BGC was normalized as N_BGC_ = F_BGC_*∗*10^6^ / ∑_total_ , where F_BGC_ is the sum of reads aligned to all biosynthetic genes in a particular BGC and ∑_total_  is the total number of reads in the metagenomic data. A BGC absent in a specific sample was assigned a value of 0.01. Spearman's rank correlation analysis was used to evaluate the correlation between BMI and the BGCs, and the p-values were corrected by the Benjamini-Hochberg method.

### 2.5. NRPS Analysis

NRPS is a class of peptide SMs produced by bacteria and fungi and has been successfully used as antibiotics [18]. AntiSMASH 4.0 was used to predict the domain information and core chemical structure of putative NRPS [17], NRPSsp was used to find the subunit of NRPS [19], and NaPDoS was used to define the class of condensation domain [20]. In order to determine the potential effect of NRPS on the obesity-related (positively or inversely) microbes, we evaluated the distribution of NRPS using the taxonomic profile of the ERP003612 data and the Human Microbiome Project (HMP) data [8, 21]. The taxonomic profiling of metagenomic reads was performed using metaphlan2 [22].

## 3. Results

### 3.1. Overview of BGCs in the Gut Microbiome of Obese Subjects

**Figure 1:**
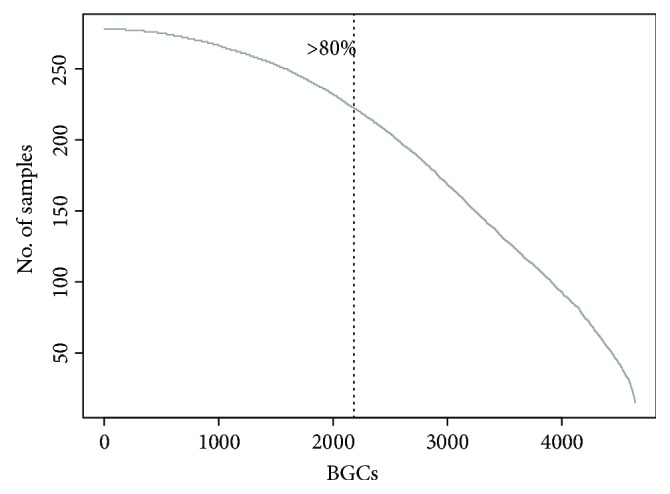
Distribution of the detected 4,640 BGCs in 278 samples.

The IMG-ABC is the largest freely accessible database of predicted and experimental BGCs that includes more than one million reads isolated from both genomes and metagenomes. After mapping the BGC reads from the obesity-related metagenomics extracted from the IMG-ABC database with the DIAMOND tool, we calculated the abundance of these BGCs by normalizing the aligned metagenomic reads from at least 10 samples with frequency of reads ≥ 10. A total of 4,640 BGCs, corresponding to ~2% of the total human-related BGCs, were finally selected, of which 2183 were detected in at least 80% of the samples (Figure 1). Most BGCs are species specific, mirroring the individual-specific taxonomic profiles of the gut microbiome [23].

**Figure 2:**
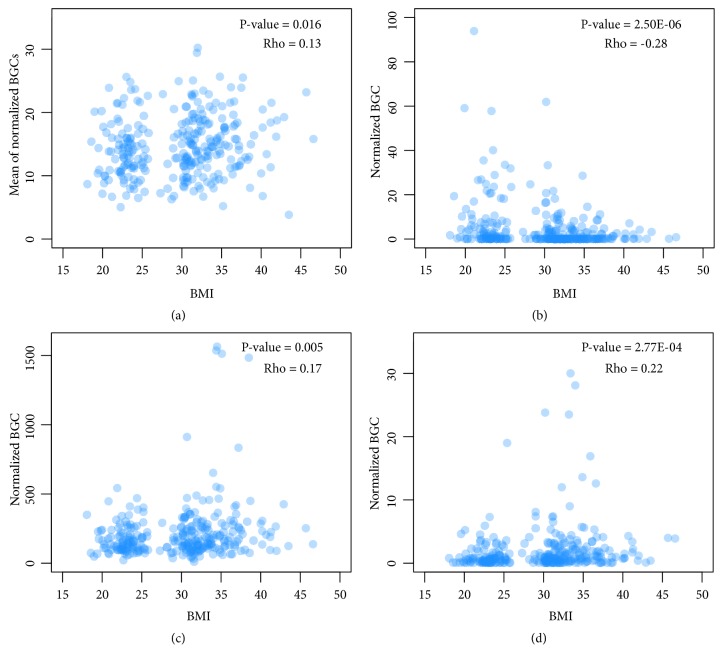
Plot showing correlation between the BMI and BGCs in 278 samples. (a) Correlation between the BMI with the mean BGC and (b, c, d) correlation of BMI with BGC 160477684, 160625038, and 160336495, respectively.

Interestingly, there was a significant positive correlation between the mean abundance of BGCs and the BMI of corresponding subjects (Figure 2(a)). The gut microbiota of obese individuals exhibited reduced taxonomic diversity compared to that of lean individuals [5]. It seems that obesity-associated SMs play a role in inhibiting the growth of competing species and reduce the diversity of the gut microbiota of obese individuals.

Spearman's rank correlation analysis was used to determine the correlation of some of the detected BGCs with BMI. The most significantly correlated BGCs are shown in Table 1, and most of them are located in metagenomic data (Genome ID with prefix “70000”), indicating that they originate from microbial species that have not been identified so far [24]. For the unknown species-derived BGCs, we defined the putative host organism by the best hit genome using NCBI-BLAST [13], and, not surprisingly, most of them belonged to obese-related genera like* Akkermansia*,* Ruminococcus*,* Bacteroides*, and* Prevotella* [4, 7, 25]. However, the association between the BGCs and BMI did not do that between the host and obesity. For example,* Bacteroides* and* Prevotella* were positively associated with BMI (Table 1), but these two genera are usually negatively associated with obesity [26]. Furthermore, many species listed in Table 1 have not been linked with obesity, e.g., Gastranaerophilaceae MH_37 (Figure 2(b)).

### 3.2. Obese Individuals Have Characteristic BGCs with Known SM

We also determined the correlation between obesity and BGCs encoding known SMs using Spearman's rank correlation analysis (Table 2). Menaquinone produced by* Enterobacter cloacae* showed the strongest correlation to BMI (Figure 2(c)). This is consistent with the high concentration of menaquinone detected in the adipose tissues of obese adults [27]. In addition,* Enterobacter cloacae *B29 isolated from the gut of morbidly obese individuals induced obesity in germfree mice [28], and reduction in* Enterobacteriaceae* and other bacteria could decrease fecal levels of menaquinone [29]. Taken together, our approach can identify obesity-associated BGCs and SMs.

### 3.3. A NRPS Found Increased with BMI

**Figure 3:**
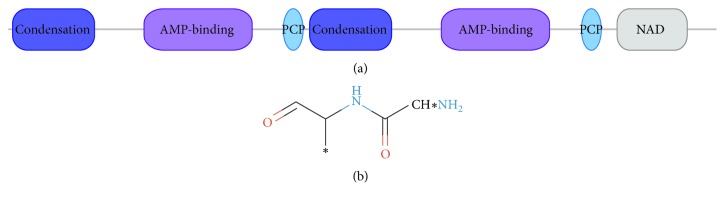
The detailed annotation of the NRPS by antiSMASH. (a) Domain annotation. (b) Predicted core chemical structure based on assumed PKS/NRPS collinearity.

We also detected an NRPS-encoding BGC (Cluster ID: 160336495) that was significantly correlated with BMI (Figure 2(d)). The best match genome of this NRPS is* Clostridium leptum* DSM 753, which is associated with both obesity and weight loss [30]. The structure of this NRPS was analyzed by antiSMASH (Figure 3), and its putative substrate was identified as phenylalanine by NRPSsp. Finally, both condensation domains of this NRPS were recognized as being of the LCL class by NapDoS.

We compared the NRPS with each phylum in the ERP003612 data and found that the Acidobacteria, Bacteroidetes, and Chlorobi were negatively associated with the abundance of this NRPS (Table 3). To further determine the potential role of this NRPS on gut microbiome of obese individuals, we calculated its abundance in HMP data and correlated it with each phylum per sample (Table 4). The phyla Bacteroidetes and Verrucomicrobia were negatively associated with the abundance of this NRPS.

## 4. Discussion

We identified several obesity-associated BGCs by comparing the metagenomics data of obese and lean individuals. In agreement with previous studies [31], the BGCs were highly host specific, with only half of them detected in at least 80% of the individuals. In addition, most of these BGCs encode for unknown secondary metabolites, thereby indicating a potential source for antimicrobials. Studies have largely focused on the effects of externally administered drugs on the human body [32], and those of endogenously produced antibiotics are virtually unknown. We found that obesity, measured in terms high BMI, was associated with increased BGC abundance, indicating lower complexity of the gut microbiome due to the inhibitory function of encoded SMs. This is consistent with a previous study which identified decreased microbial complexity of the gut as one of the factors promoting obesity [5]. This could be due to obesity-associated SMs that kill the competing bacteria and reduce diversity. In addition, an obesity associated NRPS identified in this study was negatively correlated with Bacteroidetes, an obesity-associated bacterial phylum, in both ERP003612 data and HMP data [4].

Comparison of the BGCs with known SMs between obese and nonobese individuals showed the strongest correlation of menaquinone with high BMI. In addition, many obesity-associated BGCs encoded for SMs hitherto unrelated to obesity, indicating potential biomarkers for obesity. Several BGCs are associated with mobile genetic elements like transposons that are involved in horizontal gene transfer [33] and can account for their spread across multiple genomes. This could explain the correlation of these BGCs with even the bacteria not associated with obesity. For example, the role of Bacteroidetes in obesity has been largely ambiguous [4], whereas we found an inversely association of some BGCs from this phylum with BMI. It is possible that only some species of Bacteroidetes are associated with obesity, while the rest have gained BGCs encoding for SMs that inhibit the obesity-associated species. In addition, the* Clostridium *genus of the phylum Firmicutes has been positively associated with obesity [34], especially the pathogenic* C. difficile *[35]. However, some species like* C. bolteae* [7] are more abundant in lean individuals.* Faecalibacterium prausnitzii* is another obesity-related member of family Clostridiaceae and can decrease adipose tissue inflammation and improve hepatic health [36].

To summarize, we identified obesity-related BGCs from metagenomics data and provided novel insights into gut microbial SMs as potential markers for obesity.

## 5. Conclusions

We identified 4640 BGCs in the human gut microbiota, which provides novel insights into the role of the intestinal microbial community in obesity.

## Figures and Tables

**Table 1:** Description of the top 25 correlated BGCs with BMI (p-value <0.001).

Genome ID	BGC ID	Best hit genome	P-value	Rho
2522572068	160477684	*Gastranaerophilaceae MH_37*	2.50E-06	-0.28
7000000081	161367537	*Gastranaerophilaceae Zag_1*	4.19E-05	-0.24
7000000111	161369749	*Lachnospiraceae bacterium *sp.* 8_1_57FAA*	1.51E-04	0.23
646206266	160358802	*Bacteroides *sp.* 2_2_4*	1.65E-04	0.22
7000000093	161368122	*Lachnoclostridium fimetarium DSM 9179*	1.94E-04	-0.22
7000000063	161366183	*Alloprevotella tannerae ATCC 51259*	2.03E-04	0.22
7000000036	161364337	*Proteiniborus ethanoligenes DSM 21650*	2.24E-04	-0.22
7000000308	161383461	*Bacteroides dorei CL03T12C01*	2.44E-04	0.22
641380427	160336495	*Clostridium leptum DSM 753*	2.77E-04	0.22
7000000532	161400499	*Ruminiclostridium cellobioparum termitidis CT1112*	3.95E-04	-0.21
7000000716	161411607	*Prevotella melaninogenica ATCC 25845*	4.81E-04	0.21
7000000532	161400549	*Clostridium cellulovorans 743B, ATCC 35296*	5.37E-04	-0.21
7000000172	161373025	*Clostridium *sp.* DSM 8431*	5.66E-04	-0.21
7000000187	161374310	*Ruminococcaceae bacterium AP7*	5.67E-04	-0.21
7000000666	161408821	*Prevotella melaninogenica ATCC 25845*	6.76E-04	0.20
7000000171	161372603	*Anaerotruncus rubiinfantis MT15*	7.48E-04	-0.20
7000000332	161385164	*Ruminococcus bromii L2-63*	8.60E-04	0.20
7000000579	161403195	*Barnesiella intestinihominis YIT 11860*	8.77E-04	0.20
7000000100	161368698	*Bacteroides vulgatus NLAE-zl-G202*	8.78E-04	0.20
7000000333	161385387	*Clostridium *sp.* Marseille-P299*	8.85E-04	-0.20
7000000213	161376660	*Bacteroides vulgatus mpk*	9.34E-04	0.20
7000000417	161390983	*Akkermansia muciniphila ATCC BAA-835*	9.37E-04	-0.20
7000000549	161401581	*Barnesiella intestinihominis YIT 11860*	9.43E-04	0.20
2548876788	160755921	*Bacteroides faecis MAJ27*	9.75E-04	0.20
7000000624	161405348	*Gastranaerophilaceae Zag_1*	9.92E-04	-0.20

**Table 2:** Correlation of all known SM-encoding BGCs detected in this study with BMI.

Genome ID	BGC ID	Genome description	SM	P-value	Rho
651717061	160625038	*Enterobacter cloacae*	Menaquinone	0.005	0.17
651716797	160624794	*Escherichia coli*	Yersiniabactin	0.045	0.12
2582581495	160962548	*Azospirillum brasilense Cd*	L-Rhamnose	0.052	0.12
2582581623	160962746	*Burkholderia glumae *	Toxoflavin	0.106	0.10
651716745	160624742	*Escherichia coli*	Lipopolysaccharide	0.149	0.09
2582581704	160962493	*Pasteurella multocida *	CHEBI:59393	0.177	0.08
651717167	160625143	*Lactobacillus plantarum *	L-Citrulline	0.194	-0.08
651717142	160625119	*Streptomyces chattanoogensis*	Pimaricin	0.229	0.07
651716887	160624883	*Salmonella enterica *	GDP-mannose	0.239	0.07
2563366797	160938787	*Escherichia coli KTE111*	Enterochelin	0.472	0.04
651716864	160624860	*Escherichia coli*	Lipopolysaccharide	0.587	0.03
651716612	160624612	*Escherichia coli*	Lipopolysaccharide	0.639	0.03
2582581365	160962702	*Klebsiella pneumoniae *	Lipopolysaccharide	0.655	-0.03
2563366674	160938831	*Aneurinibacillus migulanus *	GRAMICIDIN	0.981	0.00

**Table 3:** Table showing the correlation between the NRPS with each phylum in the ERP003612 data.

Phylum	P-value	FDR	Rho
Acidobacteria	1.74E-05	1.13E-04	-0.25
Actinobacteria	1.20E-11	1.56E-10	0.39
Bacteroidetes	6.60E-04	1.72E-03	-0.2
Candidatus Saccharibacteria	0.013	0.024	0.15
Chlorobi	3.17E-03	6.87E-03	-0.18
Deinococcus-Thermus	1.86E-04	8.06E-04	-0.22
Firmicutes	0.065	0.106	0.11
Fusobacteria	0.412	0.524	-0.05
Proteobacteria	0.755	0.755	0.02
Spirochaetes	0.648	0.702	-0.03
Synergistetes	0.444	0.525	0.05
Tenericutes	0.345	0.498	-0.06
Verrucomicrobia	3.36E-04	1.09E-03	0.21

**Table 4:** Table showing the correlation between the NRPS with each phylum in the HMP data.

Phylum	P-value	FDR	Rho
Acidobacteria	0.090	0.106	0.07
Actinobacteria	2.20E-16	9.53E-16	0.35
Bacteroidetes	2.20E-16	9.53E-16	-0.52
Candidatus Saccharibacteria	1.85E-05	4.81E-05	0.16
Chloroflexi	0.481	0.521	0.03
Deinococcus-Thermus	0.858	0.858	-0.01
Firmicutes	1.10E-03	2.05E-03	0.12
Fusobacteria	3.14E-04	6.81E-04	0.14
Proteobacteria	6.34E-07	2.06E-06	0.19
Spirochaetes	0.028	0.045	0.08
Synergistetes	0.044	0.057	0.08
Tenericutes	0.035	0.051	0.08
Verrucomicrobia	2.20E-16	9.53E-16	-0.33

## Data Availability

The data used to support the findings of this study are included within the article.
